# ML-based sequential analysis to assist selection between VMP and RD for newly diagnosed multiple myeloma

**DOI:** 10.1038/s41698-023-00385-w

**Published:** 2023-05-20

**Authors:** Sung-Soo Park, Jong Cheol Lee, Ja Min Byun, Gyucheol Choi, Kwan Hyun Kim, Sungwon Lim, David Dingli, Young-Woo Jeon, Seung-Ah Yahng, Seung-Hwan Shin, Chang-Ki Min, Jamin Koo

**Affiliations:** 1 Catholic Research Network for Multiple Myeloma, Catholic Hematology Hospital, College of Medicine, The Catholic University of Korea, Seoul, 06591 Republic of Korea; 2 Department of Hematology, Seoul St. Mary’s Hospital, The Catholic University of Korea, Seoul, 06591 Republic of Korea; 3 Department of Otorhinolaryngology, GangNeung Asan Hospital, University of Ulsan College of Medicine, Gangneung-si, Gangwon-do 25440 Republic of Korea; 4 Department of Internal Medicine, Seoul National University College of Medicine, Seoul National University Hospital, Seoul, Republic of Korea; 5 ImpriMedKorea, Inc., Seoul, 08507 Republic of Korea; 6 ImpriMed, Inc., Palo Alto, CA 94303 USA; 7 Division of Hematology, Mayo Clinic, Rochester, MN 55905 USA; 8 Department of Hematology, Yeoido St. Mary’s Hospital, College of Medicine, The Catholic University of Korea, Seoul, 07345 Republic of Korea; 9 Department of Hematology, Incheon St. Mary’s Hospital, College of Medicine, The Catholic University of Korea, Incheon, 22711 Republic of Korea; 10 Department of Hematology, Eunpyeong St. Mary’s Hospital, College of Medicine, The Catholic University of Korea, Seoul, 03312 Republic of Korea; 11 Department of Chemical Engineering, Hongik University, Seoul, 04066 Republic of Korea

**Keywords:** Myeloma, Translational research, Chemotherapy, Mathematics and computing

## Abstract

Optimal first-line treatment that enables deeper and longer remission is crucially important for newly diagnosed multiple myeloma (NDMM). In this study, we developed the machine learning (ML) models predicting overall survival (OS) or response of the transplant-ineligible NDMM patients when treated by one of the two regimens—bortezomib plus melphalan plus prednisone (VMP) or lenalidomide plus dexamethasone (RD). Demographic and clinical characteristics obtained during diagnosis were used to train the ML models, which enabled treatment-specific risk stratification. Survival was superior when the patients were treated with the regimen to which they were low risk. The largest difference in OS was observed in the VMP-low risk & RD-high risk group, who recorded a hazard ratio of 0.15 (95% CI: 0.04–0.55) when treated with VMP vs. RD regimen. Retrospective analysis showed that the use of the ML models might have helped to improve the survival and/or response of up to 202 (39%) patients among the entire cohort (*N* = 514). In this manner, we believe that the ML models trained on clinical data available at diagnosis can assist the individualized selection of optimal first-line treatment for transplant-ineligible NDMM patients.

## Introduction

Multiple myeloma (MM) is a hematologic malignancy of abnormal clonal plasma cells in bone marrow with the potential for uncontrolled growth, causing anemia, infections, renal impairment, and/or bone destruction. MM takes up about 1% and 10% of all cancers and hematologic malignancies, respectively. Global incidence rose 126% from 1990 to 2016, owing to an aging population and increased age-specific incidence rates^1,2^. Evolved from the conventional standard treatment of high-dose melphalan with autologous stem cell transplant (ASCT), the widespread use of proteasome inhibitors and immunomodulatory drugs, as well as the introduction of monoclonal antibodies, have made continuous improvement in the treatment and survival of MM^3,4^. A recent long-term follow-up analysis of newly diagnosed MM (NDMM) patients treated between 2007 and 2016 reported a median overall survival (OS) of 127 months, which is far superior to 30 months prior to the year 2000^5,6^. However, MM still remains mostly an incurable genetically complex disease with invariable relapse.

Relapsed MM becomes increasingly refractory to the currently available drugs. Most patients eventually succumb to complications of the relapsed, refractory disease with a dismal prognosis^7,8^. Since the duration of remission decreases with the increasing number of salvage treatments, an initial optimal choice among therapeutic armamentariums with deeper and longer remission is crucially important for MM. The current approach to treatment decision-making for NDMM takes into account a multitude of factors such as frailty, eligibility of ASCT, comorbidities, and disease subtype^9^. While the numerous clinical trials with different drug combinations provide a reference for treatment selection, cohort heterogeneity and increasing numbers make it difficult to rationally select the most adequate therapy for each patient^10^. Therefore, there has been a growing, unmet need for a technology predicting treatment outcomes at the time of diagnosis in order to adopt personalized therapeutic approaches.

The recent advances in machine learning (ML) methods led to applications in developing personalized treatment strategies for various diseases, including MM^11–14^. Algorithms such as Gaussian process regression and Random Forest have been applied to the datasets that consist of baseline clinical, biochemical, and/or gene expression data. Kubasch et al., for example, showed that the trained ML model based on Gradient Boosting Classification could predict early relapse of NDMM with 73% accuracy using four features like the first-year best response after frontline treatment^12^. Orgueira et al. reported the ML-based personalized prediction of OS for six first-line treatments using 50 variables consisting of age, International Staging System (ISS) stage, serum β_2_-microglobulin level, type of the first-line therapy, and the expression of 46 genes^13^. These previous works demonstrated the versatile applicability of ML methods in predicting clinical outcomes of multiple treatment regimens. However, most studies are based on aggregate gene aberration and/or expression data that require substantial cost and time, making it difficult to apply extensively in clinical practice. Moreover, the excessively large number of input variables used to train the models brings forth the risk of overfitting the data^15^.

The treatment regimen consisting of bortezomib, melphalan, and prednisolone (VMP) has been adopted in many countries for ASCT-ineligible NDMM patients who are not fit for intensive treatment such as quadruplet regimen^16,17^. Lenalidomide plus dexamethasone (RD) has also been used in frail and/or elderly ASCT-ineligible NDMM patients for better tolerability^18,19^. Although other quadruplet or triplet regimens such as daratumumab-bortezomib-melphalan-prednisolone (DaraVMP), bortezomib-lenalidomide-dexamethasone (VRD), daratumumab-lenalidomide-dexamethasone (DaraRD) would be considered in physically fit and transplant-ineligible NDMM patients, either VMP or RD is still regarded as an important option for frail NDMM patients^20^. To date, selection between VMP and RD regimens for NDMM has been largely dependent on the physician’s discretion as no reliable method is available for predicting each patient’s response and/or survival specific to the choice of regimen. In this study, we developed the ML-assisted methodology to address this unmet need by providing personalized predictions on the response and survival of the NDMM patients subject to VMP or RD as a first-line treatment. We first developed the ML models that can predict the OS when treated with VMP or RD regimen. We applied these ML survival models to stratify each patient into the combinatorial risk subgroups and show that an optimal choice may exist for the majority of the patients. For the patients predicted to have the same risk in both regimens, we developed the ML models that provide additional personalized prediction on response to each regimen. We explain how the ML response models can further assist with treatment selection for achieving maximal response.

## Results

### Patient characteristics

**Fig. 1:**
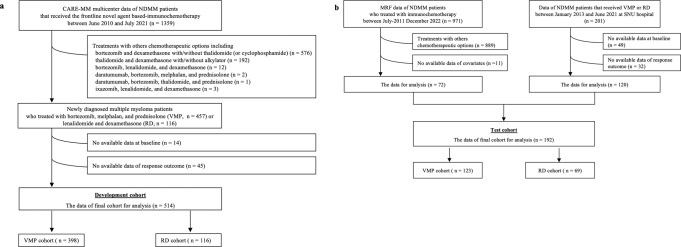
Study profile. Flow diagram describing the construction of **a** the development and **b** test cohort.

The description of the cohorts (Fig. 1) can be found in Table 1. All patients of the Catholic Research Network for MM (CARE-MM) cohort used to train the ML models were Asian. The majority of the MM Research Foundation (MMRF) cohort was white (83%) or black (15%), while the Seoul National University (SNU) cohort was all Asian. The median age at the time of diagnosis was similar among the three cohorts. The median OS of the patients treated with VMP or RD regimen in the CARE-MM cohort were 95 and 100 months, respectively; the values were 67 and 62 months among the test cohort (MMRF and SNU) for the two regimens. The overall response rates were 86 vs. 91% for the VMP and RD regimen; the rates were similar among the test cohort (87 vs. 86%). The median progression-free survival (PFS) of the patients treated with VMP or RD regimen were 15 and 21 months, respectively. The PFS lasted 24 or 40 months for the NDMM in the test cohort treated by the two regimens.

**Table 1:** Patient demographics and clinical characteristics.

Variables	Development cohort	Test cohort		
	CARE-MM (*N* = 514, %)^a^	Total (*N* = 192, %)^a^	MMRF (*N* = 72, %)^a^	SNU (*N* = 120, %)^a^
*Age, years, median (range)*	70 (40–92)	72 (57–89)	75 (57–88)	72 (63–89)
≤65	31 (6)	23 (12)	15 (21)	8 (7)
>65	483 (94)	169 (88)	57 (79)	112 (93)
*Ethnicity*				
Asian	514 (100)	121 (63)	1 (1)	120 (100)
White	0 (0)	60 (31)	60 (83)	0 (0)
Black	0 (0)	11 (6)	11 (15)	0 (0)
*Sex*				
Male	255 (50)	108 (56)	41 (57)	67 (56)
Female	259 (50)	84 (44)	31 (43)	53 (44)
*Isotype*				
IgG	273 (53)	113 (59)	43 (60)	70 (58)
IgA	108 (21)	31 (16)	11 (15)	20 (17)
Light chain	108 (21)	34 (18)	9 (13)	25 (21)
Others	25 (5)	2 (1)	1 (1)	1 (1)
Unknown	0 (0)	12 (6)	8 (11)	4 (3)
*ISS staging*				
I	118 (23)	44 (23)	19 (26)	25 (21)
II	213 (41)	62 (32)	26 (36)	36 (30)
III	168 (33)	56 (29)	21 (29)	35 (29)
Unknown	15 (3)	30 (16)	6 (9)	24 (20)
*R-ISS staging*				
I	43 (8)	35 (18)	20 (28)	15 (13)
II	299 (58)	103 (54)	34 (47)	69 (57)
III	69 (14)	27 (14)	11 (15)	16 (13)
Unknown	103 (20)	27 (14)	7 (10)	20 (17)
*High risk CA by FISH*				
Yes	91 (18)	45 (23)	22 (31)	23 (19)
No	258 (50)	102 (53)	22 (31)	80 (67)
Unknown	103 (20)	45 (23)	28 (38)	17 (14)
*Laboratory findings at diagnosis, median (range; missing %)*				
Lactate dehydrogenase, U/L	389 (98–2875; 3%)	171 (66–603; 27%)	161 (66–603; 26%)	176 (78–404; 27%)
β_2_-microglobulin, μg/mL	3.8 (0.3–50.0; 4%)	4.2 (0.2–37.7; 11%)	4.2 (0.2–17.5; 8%)	4.0 (0.8–37.7; 13%)
Albumin, g/dL	3.5 (1.2–6.4; 0%)	3.5 (1.7–5.0; 2%)	3.5 (1.7–4.7; 3%)	3.5 (2.1–5.0; 1%)
Creatinine, mg/dL	1.1 (0.4–16.2; 0%)	1.0 (0.5–11.7; 1%)	1.0 (0.5–8.8; 1%)	1.0 (0.5–11.7; 0%)
Calcium, mg/dL	9.1 (3.3–16.2; 1%)	9.3 (5.1–16.0; 1%)	9.4 (5.1–12.6; 1%)	9.1 (7.3–16.0; 1%)
Hemoglobin, g/dL	9.8 (2.9–15.9; 0%)	10.2 (6.0–16.1; 0%)	10.8 (6.8–16.1; 0%)	9.9 (6.0–16.0; 0%)
Platelets, 10^9^/L	194 (23–529; 1%)	208 (54–658; 0%)	220 (83–658; 0%)	200 (54–510; 0%)
Follow-up months, median (95% CI)	33 (30–36)	57 (48–69)	58 (53–66)	56 (45–72)

*CARE-MM* Catholic Research Network for Multiple Myeloma, *MMRF* Multiple Myeloma Research Foundation, *SNU* Seoul National University, *IgG* immunoglobulin G, *IgA* immunoglobulin A, *ISS* International Staging System, *R-ISS* Revised-ISS, *CA* cytogenetic abnormality, *FISH* fluorescent in situ hybridization.

^a^Values are numbers and percentages unless noted otherwise.

### ML-based OS prediction and risk stratification

**Fig. 2:**
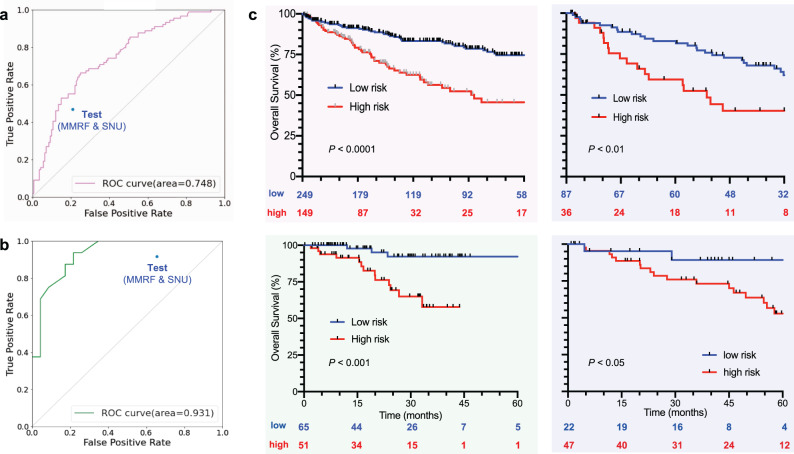
ML survival models and treatment-specific risk stratification. **a** ROC–AUC of the ML survival models predicting OS (*t* = 36 months) of the transplant-ineligible NDMM patients who received VMP or **b** RD regimen as the first-line treatment. **c** OS by the risk stratification based on the ML survival models (VMP- and RD-treated at the top and bottom, respectively). The left and right figures represent the results from the development vs. test cohort, respectively. The accuracies of the models, when applied to the independent test cohort, are marked as a dot in each graph.

We first developed the ML model for predicting the OS of the patients treated with frontline VMP. Six covariates—diabetes, lactase dehydrogenase (LDH), serum kappa, creatinine, *t*(4;14), and *t*(11;14)—were utilized to predict OS with the receiver operating characteristic-area under the curve (ROC–AUC) of 0.75 (Fig. 2a); the accuracy was 0.71 when applied to the test cohort (*n* = 123). Using the ML model estimated survival probability, we divided the VMP-treated patients into the high-risk with low survival probability (VMP^HR^; HR, high risk) and low-risk with high survival probability (VMP^LR^; LR, low risk) subgroups (149 vs. 249 patients under the threshold probability of 0.5, CARE-MM). The median OS of the VMP^HR^ subgroup was significantly inferior to that of the VMP^LR^ subgroup (Fig. 2b): 46 months vs. not reached with the hazard ratio (HR) of 2.44 (95% CI: 1.61–3.70). An HR of 2.52 (95% CI: 1.33–4.78) was observed among the two risk subgroups within the test cohort.

The VMP^HR^ subgroup had a significantly higher proportion of ISS stage III and a lower proportion of stage I (Table 2). The proportion of the NDMM patients having *t*(4;14), del(13q), or *t*(11;14) was also significantly higher among the VMP^HR^ than the VMP^LR^ subgroup. Despite its importance in the ML model (Supplementary Fig. 1), an insignificant difference was observed with respect to the rate of diabetes, reflecting the model’s nonlinearity. 30% of the patients in the VMP^LR^ subgroup were assigned ISS Stage III, indicating that the conventional staging system is not necessarily treatment-specific. Multivariate analysis on survival (Supplementary Fig. 2) confirmed the limited utility of the conventional prognostic factors when projecting survival of the NDMM patients subject to the VMP regimen as the first-line treatment. ISS, for example, was not significantly powerful in predicting OS, creatinine ≥ 2.0 mg/dL, *β*_2_-microglobulin > 5.5 μg/mL, or high-risk cytogenetic abnormalities (CA). The following three were the only clinical characteristics significantly impacting OS: calcium ≥ 11.5 mg/dL (HR 2.63; 95% CI: 1.31–5.28), albumin ≤ 3.5 g/dL (HR 0.50; 95% CI: 0.29–0.87), and high LDH (HR 1.64; 95% CI: 1.08–2.48).

**Table 2:** Baseline clinical characteristics of the VMP-specific risk subgroups.

Baseline characteristics	VMP^HR^ (*N* = 149)	VMP^LR^ (*N* = 249)	*P*	Missing
*ISS*				11
Stage I	17 (11)	59 (24)	**	
Stage II	55 (37)	109 (44)	0.2	
Stage III	73 (49)	74 (30)	**	
*Cytogenetic abnormality*				
* t*(4;14)	22/108 (20)	14/151 (9)	*	139
del(13q)	56/116 (48)	57/165 (35)	*	117
*t*(11;14)	13/113 (12)	44/161 (27)	**	124
del(17p)	17/107 (16)	14/146 (10)	0.1	145
*t*(14;16)	3/104 (3)	5/151 (3)	0.8	143
High risk	34/105 (32)	35/152 (23)	0.1	141
Age, median (range), y	71 (41–83)	70 (32–86)	0.3	0
*Sex*				0
Male	77 (52)	123 (49)	0.7	
*Comorbidities*				
Hypertension	87 (58)	137 (55)	0.5	0
Diabetes	31 (21)	41 (16)	0.3	0
Bone lytic lesions	91 (61)	171 (69)	0.1	0
*Type of myeloma*				1
Light chain disease	40 (27)	50 (20)	0.1	
Ig G	68 (46)	133 (53)	0.1	
Ig A	32 (21)	52 (21)	0.9	
Other	8 (6)	14 (6)	0.9	
*Light chain type*				1
Kappa disease	66/148 (45)	178 (71)	***	
*Hemogram*				
Hemoglobin < 10 g/dL	100 (67)	144/248 (58)	0.07	1
Absolute neutrophil counts < 1.0 × 10^9^/L	143/148 (97)	235/237 (99)	0.07	13
Platelet counts < 75 × 10^9^/L	4 (3)	17/245 (7)	0.07	4
*Laboratory variables*				
Creatinine ≥ 2.0 mg/dL	40 (27)	42/248 (17)	*	1
Calcium ≥ 11.5 mg/dL	8 (5)	9/246 (4)	0.4	3
* β*_2_-microglobulin > 5.5 μg/mL	73/143 (51)	74/239 (31)	***	16
Albumin ≤ 3.5 g/dL	88 (59)	141 (57)	0.6	0
LDH > normal	70/147 (48)	62/239 (26)	***	12

We also developed the ML model for predicting the OS of the NDMM patients who received the RD regimen as the first-line treatment. ROC–AUC of 0.93 was achieved based on the following six covariates that are mostly different from the ones used by the VMP survival model: ISS stage, *t*(4;14), kappa, creatinine, LDH, and M protein level (Fig. 2b). The accuracy was 0.78 when applied to the test cohort (*n* = 69). When comparing the two risk subgroups divided in a manner similar to the VMP treatment group (Table 3), a significant difference was observed with respect to OS (Fig. 2c). The HR of the RD^LR^ to RD^HR^ risk group among the CARE-MM and test cohort were 0.20 (95% CI: 0.08–0.54), and 0.37 (95% CI: 0.15–0.91), highlighting the applicability of the proposed methodology. Multivariate analysis (Supplementary Fig. 2) confirmed that none of the conventional factors, such as ISS and high-risk CA, significantly impact OS of the transplant-ineligible NDMM patients receiving RD regimen as the first-line treatment.

**Table 3:** Baseline clinical characteristics of the RD-specific risk subgroups.

Baseline characteristics	RD^HR^ (*N* = 51)	RD^LR^ (*N* = 65)	*P*	Missing
*ISS*				4
Stage I	20 (39)	22 (34)	0.4	
Stage II	19 (37)	29 (46)	0.4	
Stage III	9 (18)	12 (18)	1	
*Cytogenetic abnormality*				
* t*(4;14),	4/39 (10)	3/52 (6)	0.4	25
del(13q),	18/39 (46)	20/52 (38)	0.5	25
* t*(11;14),	8/40 (20)	10/50 (20)	1	26
del(17p),	7/40 (18)	9/52 (17)	0.98	24
* t*(14;16),	1/40 (3)	1/51 (2)	0.9	25
High risk	11/40 (28)	11/52 (21)	0.5	24
Age, median (range), y	71 (55–86)	73 (64–85)	0.34	0
*Sex*				0
Male	25 (49)	30 (46)	0.8	
*Comorbidities*				
Hypertension	28/50 (56)	38 (58)	0.8	1
Diabetes	11/50 (22)	16 (25)	0.8	1
Bone lytic lesions	33 (65)	47 (72)	0.4	0
*Type of myeloma*				0
Light chain disease	6 (12)	11 (17)	0.5	
Ig G	34 (67)	37 (57)	0.3	
Ig A	9 (18)	15 (23)	0.5	
Other	2 (4)	2 (3)	0.8	
*Light chain type*				2
Kappa disease	14/50 (28)	51 (80)	***	
*Hemogram*				
Hemoglobin < 10 g/dL	34 (67)	35 (54)	0.2	0
Absolute neutrophil counts < 1.0 × 10^9^/L	3 (6)	3 (5)	0.8	0
Platelet counts < 75 × 10^9^/L	2 (4)	1 (2)	0.5	0
*Laboratory variables*				
Creatinine ≥ 2.0 mg/dL	2 (4)	3 (5)	0.9	0
Calcium ≥ 11.5 mg/dL	5 (10)	1 (2)	0.05	0
* β*_2_-microglobulin > 5.5 μg/mL	8/48 (17)	12 (19)	0.8	5
Albumin ≤ 3.5 g/dL	26 (51)	31 (48)	0.8	0
LDH > normal	14 (27)	11 (17)	0.2	0

**Fig. 3:**
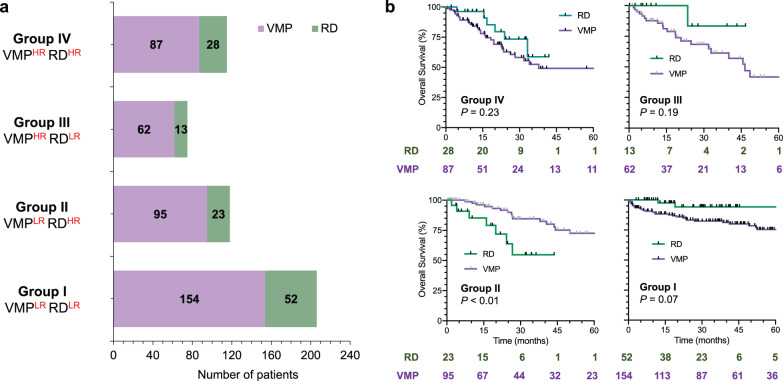
Combinatorial risk stratification of the development cohort with respect to VMP and RD regimen. **a** Number of NDMM patients in the four combinatorial risk subgroups. **b** OS of the patients in each subgroup treated by VMP or RD regimen.

We next classified the entire cohort into the four combinatorial risk subgroups using the two ML survival models (Fig. 3). Among the total of 514 transplants ineligible NDMM patients (CARE-MM), 40% belonged to Group I (VMP^LR^RD^LR^ risk); 23% belonged to the Group II (VMP^LR^RD^HR^ risk); 15 and 22% belonged to Group III (VMP^HR^RD^LR^ risk) and IV (VM^HR^RD^HR^ risk), respectively (Fig. 3a). Survival analysis (Fig. 3b) showed that the median OS of the Group IV was 62 months, which is markedly inferior to ≥120 months of the Group I. Notably, OS of the patients treated with VMP regimen in the Group II was significantly superior to that of those treated with RD regimen (HR of 0.15; 95% CI: 0.04–0.55). In Group III, the OS of the patients treated with the VMP regimen showed a superior tendency compared to those treated with the RD regimen without statistical significance (*P* = 0.19); the HR was 0.45 (95% CI: 0.13–1.50). Similar trends were also observed when applying the proposed risk stratification to the test cohort (Supplementary Fig. 3). These results show that the proposed treatment-specific risk stratification based on the ML survival models can be helpful in selecting frontline regimen for at least 85 patients (17%) among the cohort: 62 patients treated with VMP regimen in the Group III and 23 patients treated with RD regimen in the Group II patients might have experienced superior survival if treated with the unchosen ones.

### ML-based prediction of early optimal response

The ML survival models cannot discriminate between VMP and RD regimens for the NDMM patients in Group I and IV, as the insignificant difference in OS is expected. For these groups, we focused on early optimal response, which is an independent endpoint from OS based on the results of the survival and multivariate analysis (Supplementary Figs. 4 and 5). The analysis based on the development cohort revealed that the median PFS was significantly longer (*P* < 0.0001) for the early optimal responders (EOR, *n* = 132) than the suboptimal responders (ESR, *n* = 382): 25 vs. 15 months with the HR of 0.58 (95% CI: 0.46–0.74). Overall best response (OBR, Supplementary Fig. 6) was also significantly superior among the EOR; the rate of complete response (CR) in OBR was 65 vs. 17% (*P* < 0.0001) in the EOR and ESR of the development cohort, respectively. Insignificant differences with respect to OS were observed between the two types of responders. The same trends were confirmed among the test cohort (Supplementary Fig. 7).

**Fig. 4:**
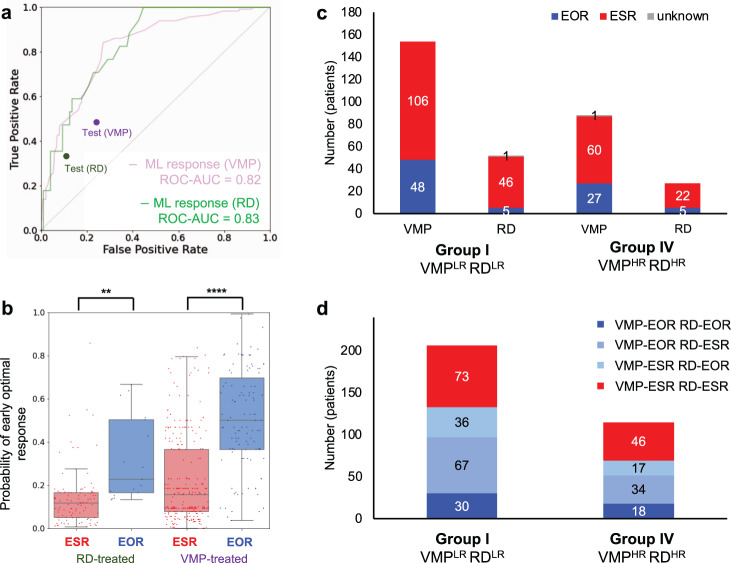
ML response models and their application to further refine treatment for the Group I and IV of the development cohort. **a** ROC–AUC of the ML response models predicting the early optimal response of the transplant-ineligible NDMM patients who received VMP or RD as the first-line treatment. **b** Distribution of the probabilities of early optimal response estimated by the ML response models. **c** Proportions of the EOR and ESR in Groups I and IV based on the conventional treatment selection. Each box plot displays the interquartile range (IQR), with the lower boundary representing the 25th percentile and the upper boundary representing the 75th percentile. The line within the box displays the median, and the whiskers extend to ±1.5 × IQR. **d** Predicted proportions of the EOR and ESR to VMP and RD based on the ML response models.

Given the clinical utility of early optimal response, we developed the ML models for predicting response outcomes for each regimen. ROC-AUC of the final ML response models were 0.82 and 0.83 for VMP and RD regimens, respectively (Fig. 4a). The accuracies were also high when tested using the MMRF and SNU cohort: 70% (VMP) and 81% (RD). The former relied on four (type of MM, del(13q), *t*(11;14), and diabetes) while five covariates (ISS, light chain, albumin level, 24 h-urine protein level, and absolute neutrophil level (ANC)) were required by the latter for computing a prediction. Predictive performance of the response models was consistently high for both regimens, generating a significantly higher probability of early optimal response for the EOR and a lower probability for the ESR (Fig. 4b and Supplementary Fig. 8). Clinical follow-up results with the conventional selection between VMP and RD regimen resulted in 26% (53/206) in the Group I and 28% (32/115) in the Group IV achieving early optimal response (Fig. 4c). However, the ML response models predicted that 65% of the Group I and 60% of the Group IV would have become EOR if treated with the regimen having a higher probability of early optimal response (Fig. 4d). Only 35 and 40% were predicted to be ESR to both regimens among the Group I and IV, respectively. In this manner, the ML response models might have contributed to better response and longer PFS of up to 117 NDMM patients in these two combinatorial risk subgroups.

## Discussion

VMP and RD regimens utilize different classes of therapeutic drugs—proteasome inhibitor vs. immunomodulator. There has not been a clinical trial comparing the two regimens head-to-head against the transplant-ineligible NDMM. Instead, only a retrospective analysis comparing the two can be found in the literature, which reported a longer PFS for the RD-treated but a significantly reduced risk of death for the VMP-treated elder NDMM^21^. RD was associated with a longer PFS among the cohort in this study, while an inconclusive difference in OS was observed (Supplementary Figs. 6 and 7).

Currently, available prognostic evaluations for NDMM patients at diagnosis, such as revised ISS (R-ISS), International Myeloma Working Group (IMWG), and prognostic nomogram, may be helpful in identifying patients requiring more aggressive therapy or intensive follow-up. These prognostic evaluations are, however, dynamic and can evolve with response to treatment; they provide insufficient information regarding the selection of an initial risk-adapted treatment for every single patient. As such, there is an increasing consensus on the need to incorporate predictive response to frontline treatment^22–27^. Gene expression profiles appear to be a plausible solution but are currently limited to use in clinical trials at tertiary (academic) centers. In this regard, real-world evidence (RWE) based models are receiving increased attention as an alternative to conventional randomized clinical trials^28,29^.

**Fig. 5:**
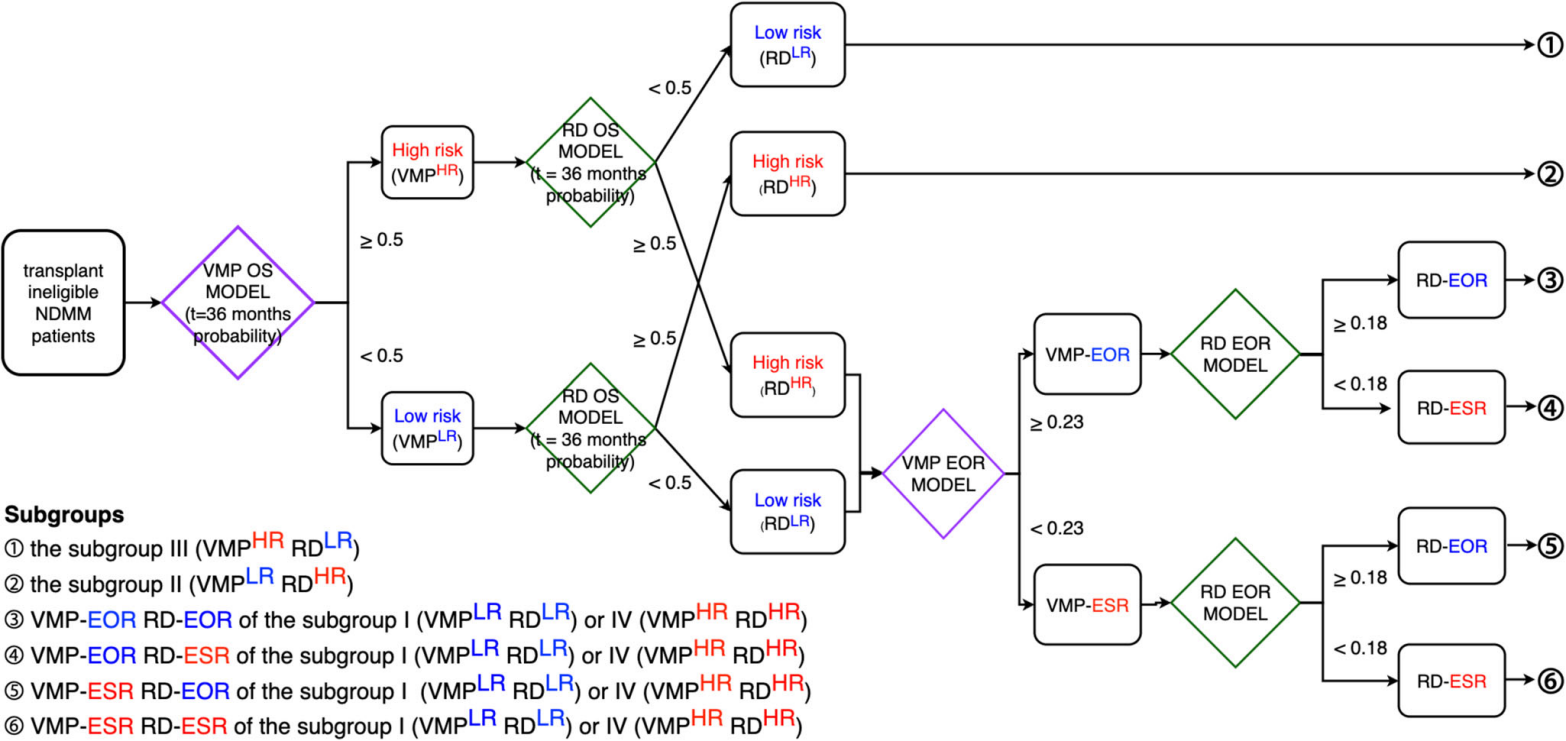
Schematic of the proposed methodology that assists treatment selection for the transplant-ineligible NDMM based on the application of ML survival and response models.

In this study, we developed ML survival and response models to assist treatment selection between VMP and RD for patients with ASCT-ineligible NDMM (Fig. 5). A total of 8 covariates—ISS, diabetes, LDH, serum kappa, creatinine, urine M protein level, *t*(4;14) and *t*(11;14)—available at diagnosis were required to compute individualized prospects on survival specific to administration of VMP or RD regimen as a first-line treatment. The survival prospects can be used to avoid regimens likely to result in early death, while the response prospects can help to select a regimen likely to achieve better response and longer PFS. Survival analysis suggested that treatment selection based on the ML survival models might help to achieve superior OS in 17% (85/514) of the cohort within Group II (VMP^LR^RD^HR^) and III (VMP^HR^RD^LR^). In addition, 39% within the Group I (VMP^LR^RD^LR^) and IV (VMP^HR^RD^HR^) might have achieved early optimal response and superior PFS by selecting the regimen based on the ML response models requiring a total of 10 covariates—ISS stage, type of myeloma, age, del(13q), t(11;14), diabetes, albumin level, creatinine level, urinary protein level, and ANC. The combined number of patients who might have benefited from the ML models is 202 (39%), which we believe is large enough to provide the foundation for a prospective study.

EOR to VMP or RD regimen in our cohort enjoyed a significantly superior rate of CR and longer PFS. An insignificant difference was observed with respect to OS, which was somewhat expected since the choice of subsequent treatments and responses can also impact survival. The most important variable for predicting the early optimal response to the VMP regimen was the type of myeloma (light chain disease, IgG, IgA, or other). IgG type MM, in particular, was the most powerful negative predictor: Only 9% of the IgG type achieved an early optimal response to the VMP regimen. While del(13q) alone was not one of the top 10 different lcharacteristics between the two types of responders (Supplementary Fig. 5), the CA resulted in the greatest improvement in ROC–AUC of the ML response model when used in addition to the type of myeloma. The same was true for t(11;14) and diabetes, which were effective in improving the predictive performance when combined with the aforementioned covariates. Del(13q) has been implicated in the regulation of the cell cycle and apoptosis. The rate of del(13q) is known to be the highest (50%) among chronic lymphoblastic leukemia (CLL) and is associated with a better prognosis^30^. A previous study based on 1181 NDMM patients reported that del(13q) was significantly associated with longer OS but not PFS, even after adjusting for the presence of *t*(11;14) and *t*(6;14)^31^. These patients received several different types of regimens as the first-line treatment, among which RD, given to less than half of the cohort, was the only same regimen when compared to this study. In this regard, the ultimate role of del(13q) and the influence of deletion size, burden, and type in association with the retinoblastoma (RB1) gene mutation need to be revealed in future studies. The other covariates, such as creatinine level and ANC, have been associated with the response and/or prognosis of NDMM patients^32,33^; therefore, it is not surprising that the proposed ML models utilize these covariates as well as the aforementioned variables.

We focused on developing ML models that utilize a minimal subset of the covariates for computing a prediction. ML methods are powerful in building and/or identifying structured relationships between features for classifying or quantifying outcome(s). ROC–AUC tends to increase as more features are available for predicting an outcome assuming that each covariate is not strongly correlated with another. Therefore, it is common to utilize the entire features, i.e., clinical characteristics and/or multi-omics data, in training the ML models for predicting drug response or survival. However, such an excessive use of features makes the models difficult to explain. The difficulty and cost of measuring and recording all data accurately increases with the number of features. The risk of overfitting the data increases as well, making the models less accurate when predicting outcomes for new cases^34,35^. In the absence of the gold standard, the number and composition of features used in the ML models are often justified based on predictive performance and data size. Among the two subsets of the development cohort, the NDMM patients treated with the VMP regimen (*N* = 398) are the largest reported to date to our knowledge and make the respective ML models more credible. The external, independent validation using the test cohort resulted in a similar level of performance.

The proposed approach and ML models have a few limitations. To begin with, the ML models were trained with the cohort who were diagnosed and treated in S. Korea. The homogeneous ethnicity (Asian) of the cohort may limit applicability, although the covariates used to train the models are universally measured during diagnosis. Although the developed ML model would be more powerful by adding submodel(s) targeting other meaningful endpoints, including adverse events, medical cost, or subjective satisfaction index, such analysis was limited due to retrospective design. The choice between VMP and RD regimen for the transplant-ineligible NDMM is also not globally applicable because new combinations, such as DaraVMP, VRD, or DaraRD, have been widely accepted around the world as the first-line treatment^36^. However, a recent abstract from the Italian phase IV study (NCT03829371)^37^ showed that making the nuanced medical decision between VMP vs. RD may still be a relevant one. For example, while in the clinical trial, 2-year OS was 89% with VMP, our ML model predicted the same OS for patients stratified as VMP^LR^. Similarly, while the randomized trial predicted a 2-year OS of 75% for patients on RD, our ML survival model predicted a 2-year OS of 70% for RD^HR^. These observations suggest that our results can be readily extrapolated, regardless of racial disparities.

Although our model could be more useful for frail NDMM patients who are not fit for the newly introduced triplet or quadruplet regimen, we could not investigate the impact of frailty due to its not being available in the datasets. We thus believe that the proposed approach utilizing the ML models to gain individualized insights on survival and response prospects is of greater value than the models themselves. Another limitation is related to drug toxicity and quality of life, which can dictate the choice of regimen but are not explicitly addressed by the ML models. In spite of these limitations, we believe that the proposed approach and ML models can assist in treatment selection for the transplant-ineligible NDMM. The models provide prospects on OS and response that are treatment- and patient-specific, enabling more refined risk stratification. The ML models can be trained and operate in real-time using RWE data and clinical characteristics measured at diagnosis, requiring minimal resources for adoption in clinical practice. We believe that our methodology can contribute to identifying an optimal first-line treatment and improving survival in ASCT-ineligible NDMM patients.

## Methods

### Patient selection and data sources of the development cohort

The clinical data used in this study were extracted from the multicenter registry database of the Catholic Research Network for MM (CARE-MM), which include four university hospitals—Seoul, Yeouido, Eunpyeong, and Incheon St. Mary’s Hospital—in the Republic of Korea. There were records of the 1359 consecutive NDMM patients who received the novel agent based-therapy as the first-line treatment between June 2010 and July 2021. Among them, 786 patients treated with options other than VMP or RD regimen were excluded. We further excluded 59 patients who did not have the baseline data or response outcome (Fig. 1a). A total of 514 patients remained to be included; 398 and 116 were treated with VMP or RD regimens, respectively. This study was reviewed and approved by the Institutional Review Board (IRB) of the Catholic University, Seoul, Republic of Korea (IRB No. XC22RIDI0026), with an informed consent waiver due to the use of retrospective clinical data.

Thirty-nine types of demographic and clinical characteristics measured at and/or obtained by the diagnosis date were retrieved from the multicenter registry database. Baseline demographics such as age and sex had no missing values, while up to 10% of the values were missing for the following: Comorbidities (hypertension, diabetics), presence of MM-related symptoms (hypercalcemia, renal impairment, anemia, bone lytic lesion, extramedullary plasmacytoma), blood cell counts, results of blood chemistry test, and ISS^38^. Revised ISS (R-ISS) assigned according to the previously reported criteria^39^ were missing for 20% of the cohort. CA including the high-risk indicators—*t*(4;14), *t*(14;16), and del(17p)—measured by fluorescent in situ hybridization, as well as 13q deletion^40^ were available for 62–67% of the cohort with the exception of 1q21 (28%).

### Patient selection and data sources of the test cohort

The independent test datasets were obtained from two different sources: CoMMpass database (version IA17) and SNU Hospital (Fig. 1b). The former is the result of the ongoing MMRF Personalized Medicine Initiatives (https://research.themmrf.org); the latter was approved by the IRB of the SNU Hospital (IRB No. H-2211-143-1381). The MMRF dataset included the consecutive 917 NDMM patients who received frontline treatment between July 2011 and December 2022; the SNU dataset consisted of consecutive 201 NDMM patients who received VMP or RD from January 2013 to June 2021. When excluding the patients with missing data on baseline and/or response variables, 72 and 120 NDMM patients treated with VMP or RD regimen as the first-line treatment remained.

### Outcomes

OS and early optimal response were the clinical endpoints for which predictive ML models were developed. None were used as features in training the ML models. The index date of both endpoints was the first day of administration of VMP or RD unless stated otherwise. The former was measured from the index date to censoring or death from any cause. The latter was defined as the achievement of complete response or very good partial response (VGPR) by the 8^th^ week since the index date; partial response, stable disease, or refractory disease were classified as an early suboptimal response. Response to each VMP or RD was evaluated using the IMWG’s response criteria^41^. PFS was calculated as the durations from the index date until progression or death.

### Development of ML models and treatment-specific risk stratification in the development cohort

The raw dataset of the development cohort contained the 39 clinical characteristics of the 514 transplant-ineligible NDMM treated by VMP or RD regimen. For the categorical variables, we applied label encoding to convert them into numerical data^42^. The rest were continuous, numerical variables, which we re-scaled using the robust scaler. Missing data were left as is, meaning the patients with missing data were still included during training since this resulted in the highest performance. We tried replacing the missing data with median and imputed values; however, the predictive performance became lower when either of the measures was taken (Supplementary Fig. 9). Imputation was attempted in two different ways—*k* nearest neighbors with *k* = 5 and the MICE (multivariate imputation by chained equation) technique^43,44^. The kNN imputation was performed using the *KNNImputer* (sklearn) while the *impyute.imputation.cs* was employed for the latter (MICE).

ML models were developed using the XGBoost (eXtreme Gradient Boosting) method^45^. From the total of 39 demographic and clinical variables, a minimal set was chosen via sequential forward feature selection^42^. As a result, no variables remained that enabled more than 0.01 improvement in ROC–AUC during validation. Using the minimal set of variables, response and survival models were trained to compute the probability of early optimal response (binary classification) or time-course of changes in the probability of being alive using accelerated failure time. Each treatment group (*N* = 398 for VMP and 116 for RD) was divided into the training and validation set during 5-fold cross-validation via the *StratifiedKFold* that minimizes the differences in the composition of classes such as EOR and ESR across the folds^42^. The following set of parameters was used to train the XGBoost model that is optimized with respect to the log loss function: *n_estimators* = 1,000 and *early_stopping_rounds* = 200. Random states ranging from 0 to 500 with increments of 10 were tested to identify the models with the highest performance in both the development and test cohort.

The predictive performance of the trained ML models was assessed with respect to the ROC–AUC recorded during internal validation in the development cohort. For the ML survival models, performance was first evaluated by using the computed probability of survival at various time points (*t* = 18, 24, 30, 36 months) to identify the optimal model. The specific time point (*t* = 36 months from the first administration of the regimen) was chosen after iteratively testing model performance with respect to ROC–AUC and the ratio of dead to alive (Supplementary Fig. 10).

The ML survival models were employed in stratifying the patients into treatment-specific risk subgroups. For the NDMM treated with the VMP regimen, we used the computed probability of survival (*t* = 36 months) during validation for the VMP-specific risk stratification, while the probability of survival following RD was calculated using the model trained with the dataset of the RD treatment group. And it was vice versa for the NDMM treated with the RD regimen. The threshold probability for each stratification was chosen between the default (0.5) and the one that maximizes the F1 score during validation based on the utility of the resultant risk stratification.

### External validation of the ML models using the test cohort

We tested the predictive performance of the ML models using the independent datasets provided by the MMRF and SNU hospitals. No modifications were made to the SNU dataset, while the following covariates of the MMRF dataset were rescaled to make the units the same: albumin (g/dL), creatinine (mg/dL), hemoglobin (g/dL), IgA level (mg/dL), and 24 h-urinary M protein level (mg/24-h). The performance of the survival models was evaluated with respect to the accuracy of predicting OS (*t* = 36 months) as well as the HR between the two risk groups. The accuracy of predicting early optimal response based on the threshold level set during training was used to assess the ML response models.

### Statistical analysis and performance evaluation

Statistical tests were conducted by using the *InStat* package (Prism). The performance of the ML models was assessed using the *roc_curve* and *auc* package (scikit-learn). Survival analysis was performed using the Kaplan-Meier method; differences in survival were tested using the log-rank test. The Cox proportional hazards model was used to compute an HR during multivariate analysis, while the associated *P* values were calculated using the likelihood method. Cox proportional hazards model analyses were performed by the *sksurv* package (scikit-learn). When analyzing the patient subgroups, categorical and continuous variables were compared using the chi-square, Fisher’s exact, *t*- or Mann–Whitney *U* test, as appropriate (Supplementary Fig. 11). Distribution of the probabilities computed by the ML models were compared using the Mann-Whitney U test. The significance level was set at *P* < 0.05. Analyses were performed using Python (version 3.9.7) or Prism 8 (GraphPad, San Diego, CA, USA).

### Reporting summary

Further information on research design is available in the Nature Research Reporting Summary linked to this article.

## Supplementary information

Supporting Information

REPORTING SUMMARY

## Data Availability

The datasets generated and/or analyzed during the current study are available from the corresponding author on reasonable request, with the exception of the MMRF dataset, for which access is granted by the MMRF.

## Code availability

Analyses were performed using Python (version 3.9.7) or Prism 8 (GraphPad, San Diego, CA, USA). The code used to analyze datasets is available from the corresponding author upon reasonable request.
