# Supplementary material for: ML-based sequential analysis to assist selection between VMP and RD for newly diagnosed multiple myeloma
Source: NPJ Precis Oncol. 2023 May 20;7:46. doi: 10.1038/s41698-023-00385-w (PMC10199943; doi:10.1038/s41698-023-00385-w)
Supplement: Supplementary file 1 — Supporting Information [file 41698_2023_385_MOESM1_ESM.pdf]

Supporting Information for

ML-based sequential analysis to assist selection between VMP and RD for newly diagnosed multiple myeloma

Sung-Soo Park[1,2,+], Jong Cheol Lee[3,+], Ja Min Byun[4,+] Kyucheol Choi[5], Kwan Hyun Kim[5], Sungwon Lim[5,6], David Dingli[7], Young-Woo Jeon[1,8], Seung-Ah Yahng[1,9], Seung-Hwan Shin[1,10], Chang-Ki Min[1,2,*], Jamin Koo[5,6,11,*]

[1]Catholic Research Network for Multiple Myeloma, Catholic Hematology Hospital, College of Medicine, The Catholic University of Korea, Seoul 06591, Republic of Korea
[2]Department of Hematology, Seoul St. Mary's Hospital, The Catholic University of Korea, Seoul 06591, Republic of Korea
[3]Department of Otorhinolaryngology, GangNeung Asan Hospital, University of Ulsan College of Medicine, Gangneung-si, Gangwon-do 25440, Republic of Korea
[4]Department of Internal Medicine, Seoul National University College of Medicine, Seoul National University Hospital, Seoul, Republic of Korea
[5]ImpriMedKorea, Inc., Seoul 08507, Republic of Korea
[6]ImpriMed, Inc., Palo Alto, CA 94303, USA
[7]Division of Hematology, Mayo Clinic, Rochester, MN, USA
[8]Department of Hematology, Yeoido St. Mary's Hospital, College of Medicine, The Catholic University of Korea, Seoul 07345, Republic of Korea
[9]Department of Hematology, Incheon St. Mary's Hospital, College of Medicine, The Catholic University of Korea, Incheon 22711, Republic of Korea
[10]Department of Hematology, Eunpyeong St. Mary's Hospital, College of Medicine, The Catholic University of Korea, Seoul 03312, Republic of Korea.
[11]Department of Chemical Engineering, Hongik University, Seoul 04066, Republic of Korea

[+] These authors contributed equally to this work.

Table of Contents

Supplementary Fig. 1. Importance of the selected covariates in the ML survival model predicting OS of the NDMM treated by (**A**) VMP or (**B**) RD regimen

Supplementary Fig. 2. Hazard ratios of the baseline clinical characteristics for OS of the NDMM treated by VMP or RD regimen as the first-line treatment

Supplementary Fig. 3. Combinatorial risk stratification of the test cohort with respect to VMP and RD regimen. (A) Number of the NDMM patients in the four combinatorial risk subgroups. (B) OS of the patients in each subgroup treated by VMP or RD regimen

Supplementary Fig. 4. Comparison of the (A) PFS and (B) OS between EOR and ESR to VMP or RD regimen

Supplementary Fig. 5. Hazard ratios of the baseline clinical characteristics for PFS of the NDMM treated by VMP or RD regimen as the first-line treatment

Supplementary Fig. 6. Clinical outcome and survival of the development cohort (CARE, N = 514). Overall response rates of the NDMM patients to (**A**) VMP or (**B**) RD regimen as the first-line treatment. (**C**) PFS and (**D**) OS of the VMP or RD treated NDMM patients.

Supplementary Fig. 7. Clinical outcome and survival of the external test cohort (MMRF & SNU, N = 192). Overall response rates of the NDMM patients to (**A**) VMP or (**B**) RD regimen as the first-line treatment. (C) PFS and (D) OS of the VMP or RD treated NDMM patients.

Supplementary Fig. 8. ML response models and its application to further refine treatment for the Group I and IV of the test cohort. (A) Proportions of the EOR and ESR in the Group I and IV based on the conventional treatment selection. (B) Predicted proportions of the EOR and ESR to VMP and RD based on the ML response models

Supplementary Fig. 9. Changes in the predictive performance of the ML survival model (VMP regimen) with respect to the handling of missing values. OS of the resultant risk stratification when the missing values were (**A**) left as is, (**B**) replaced with median, imputed using (**C**) kNN, or (**D**) MICE

Supplementary Fig. 10. Changes in the (A) ratio of dead to alive and (B) predictive performance of the ML survival models (VMP left, RD right) with respect to the time point chosen to stratify the patients into risk groups

Supplementary Fig. 11. Ranked heat map of the *P* values obtained when comparing the EOR and ESR to VMP regimen

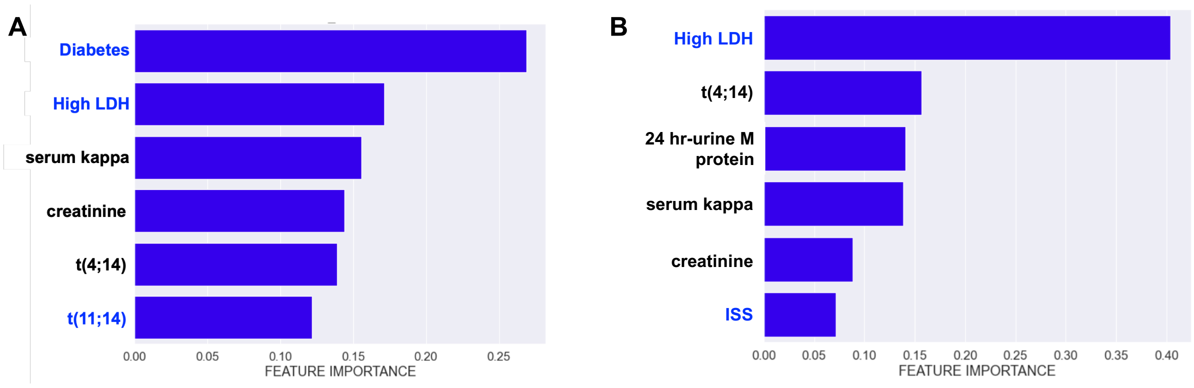

Supplementary Fig. 1. Importance of the selected covariates in the ML survival model predicting OS of the NDMM treated by (**A**) VMP or (**B**) RD regimen. Categorical covariates are written in blue while the numerical ones are written in black. High LDH is coded 0 or 1 with respect to >450 U. Feature importance was calculated based on the average gain across all splits where a feature was used.

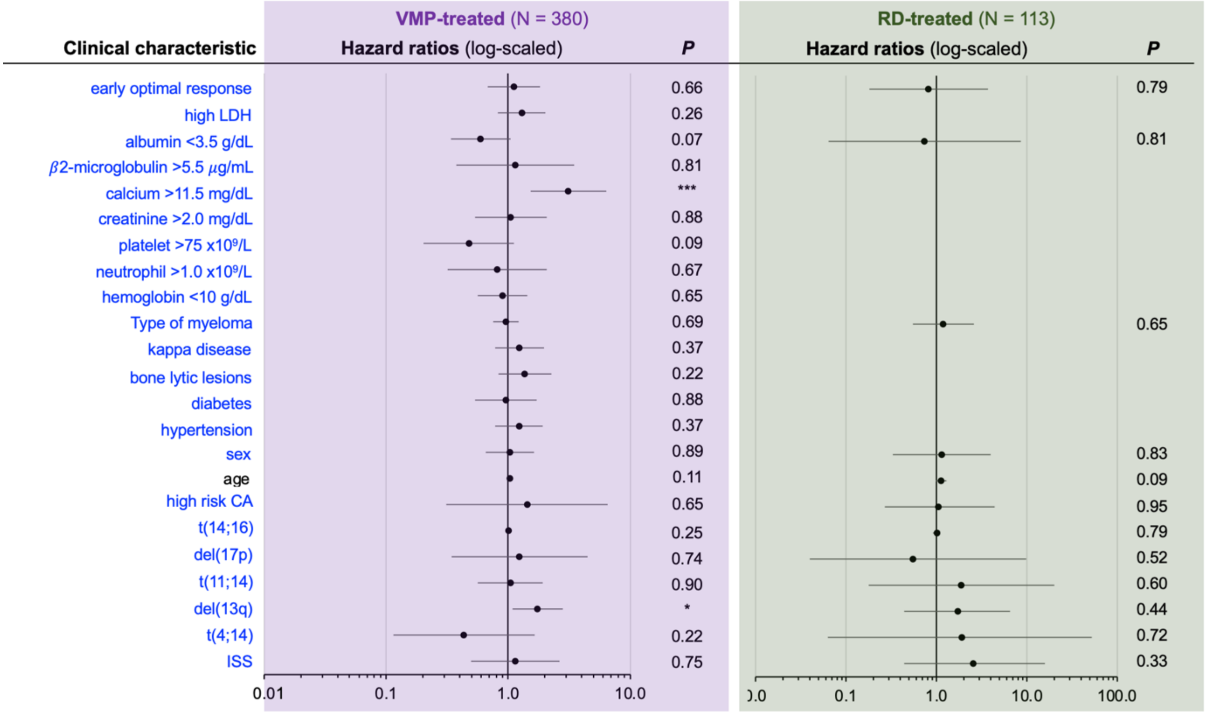

Supplementary Fig. 2. Hazard ratios of the baseline clinical characteristics for OS of the NDMM treated by VMP or RD regimen as the first-line treatment. The HR and *P* values were calculated via the Cox proportional hazard modeling. Categorical covariates are written in blue while the numerical ones are written in black. A commonly reported subset of the covariates were chosen for the multivariate analysis with respect to the RD-treated NDMM patients due to the limited number of events and patient number. The index date of PFS was the 8th week since the first administration of VMP or RD regimen.

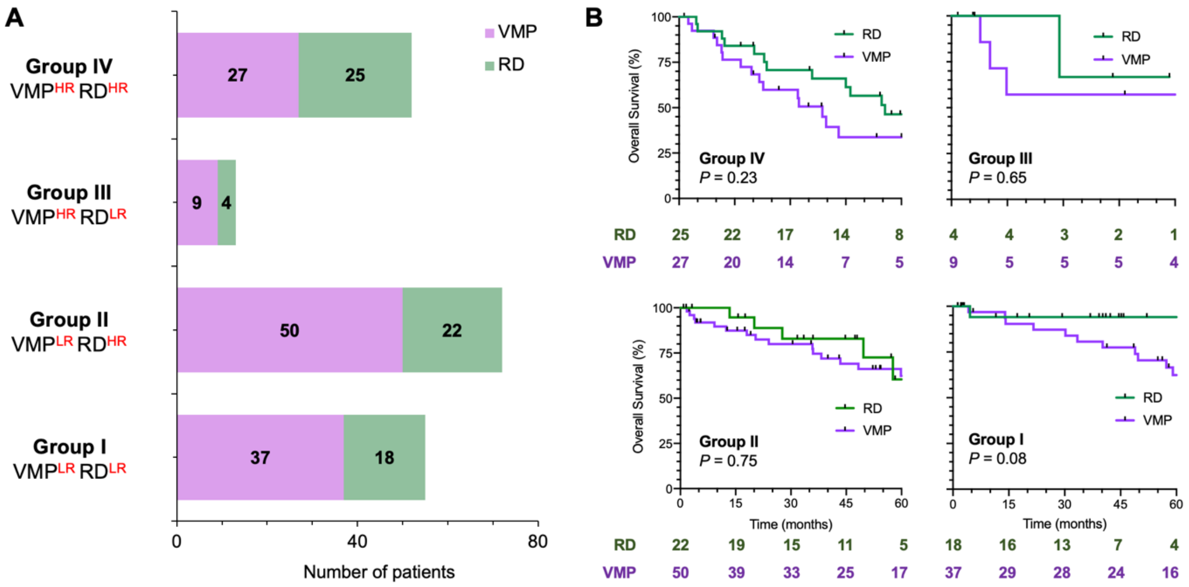

Supplementary Fig. 3. Combinatorial risk stratification of the test cohort with respect to VMP and RD regimen. (A) Number of the NDMM patients in the four combinatorial risk subgroups. (B) OS of the patients in each subgroup treated by VMP or RD regimen

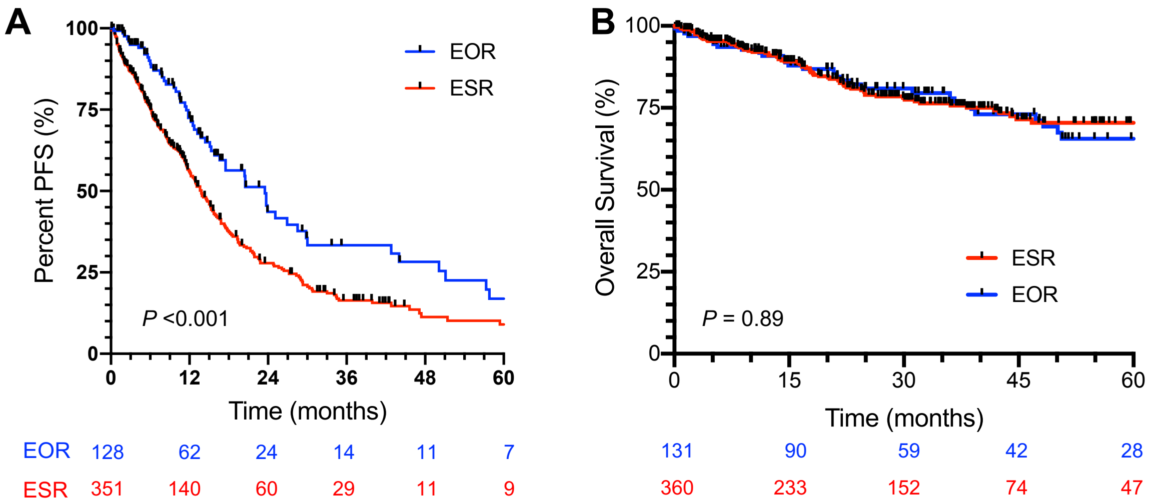

Supplementary Fig. 4. Comparison of the (**A**) PFS and (**B**) OS between EOR and ESR to VMP or RD regimen. The *P* values are calculated by the log-rank test. The index date of PFS was the 8th week since the first administration of VMP or RD regimen.

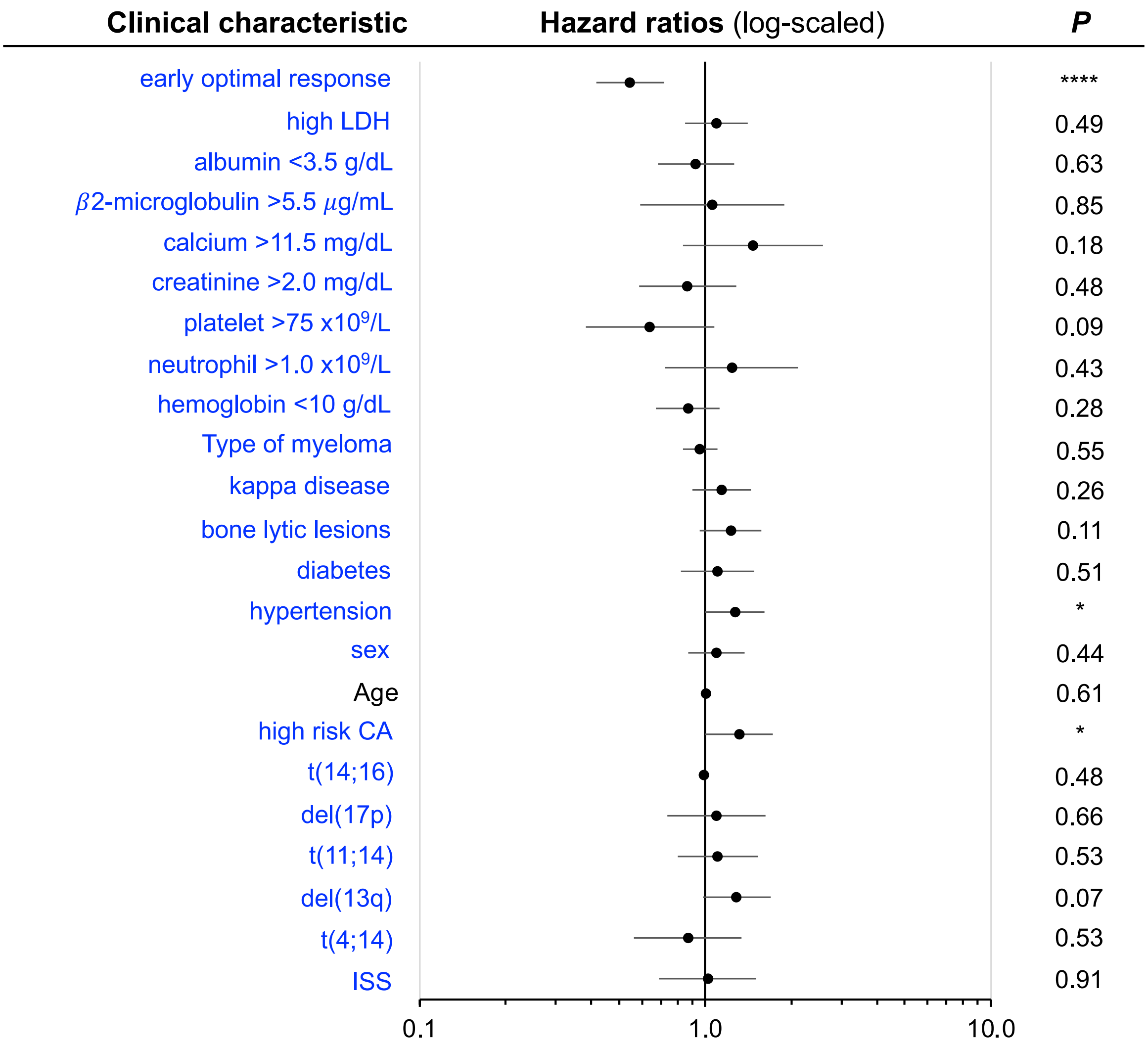

Supplementary Fig. 5. Hazard ratios of the baseline clinical characteristics for PFS of the NDMM treated by VMP or RD regimen as the first-line treatment (N = 514). The HR and *P* values were calculated via the Cox proportional hazard modeling. Categorical covariates are written in blue while the numerical ones are written in black. The index date of PFS was the 8th week since the first administration of VMP or RD regimen.

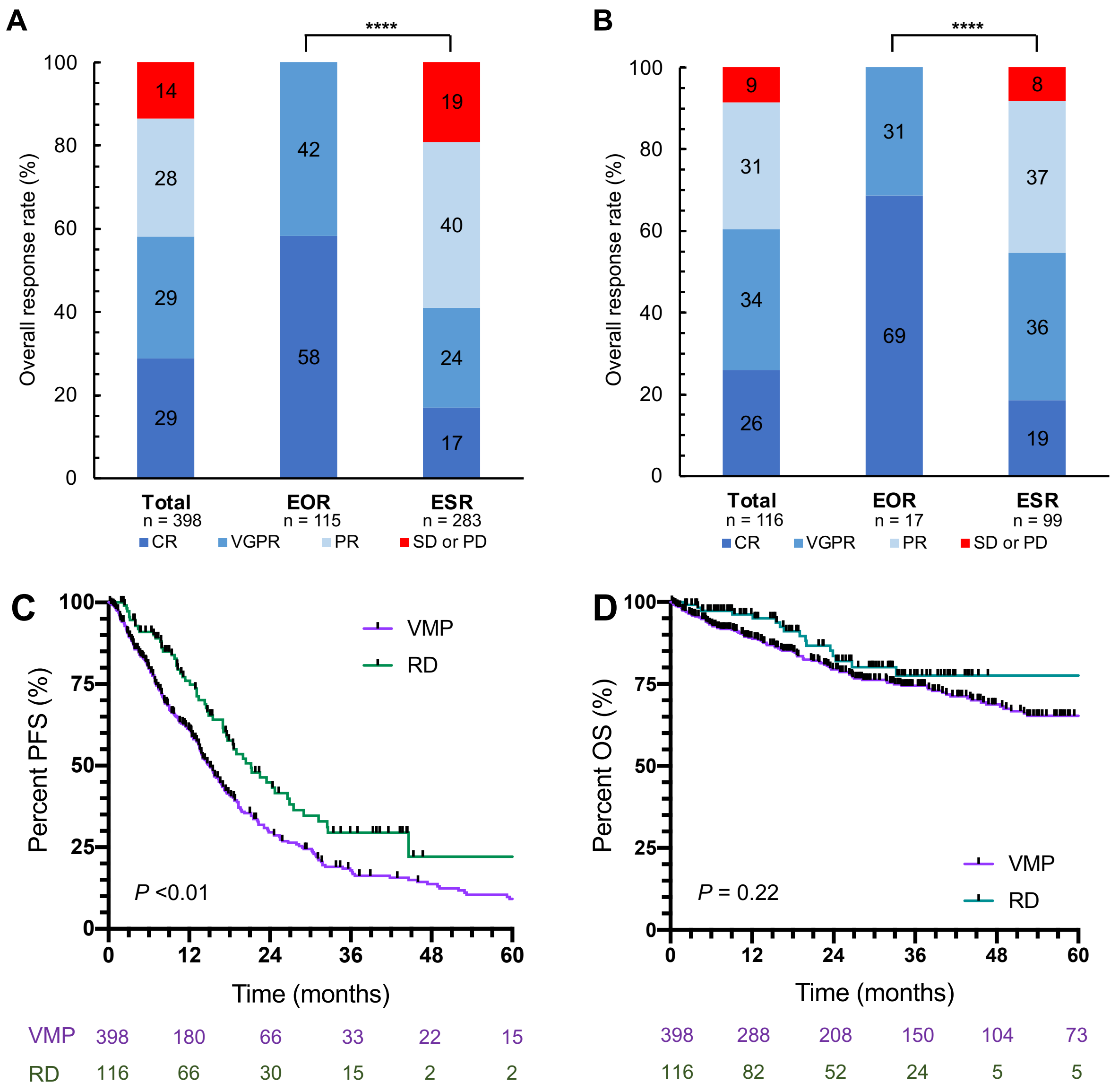

Supplementary Fig. 6. Clinical outcome and survival of the development cohort (CARE, N = 514). Overall response rates of the NDMM patients to (**A**) VMP or (**B**) RD regimen as the first-line treatment. The *P* values are calculated using the chi-square and Fisher's exact test, respectively. (**C**) PFS and (**D**) OS of the VMP or RD treated NDMM patients. The *P* values are calculated by the log-rank test.

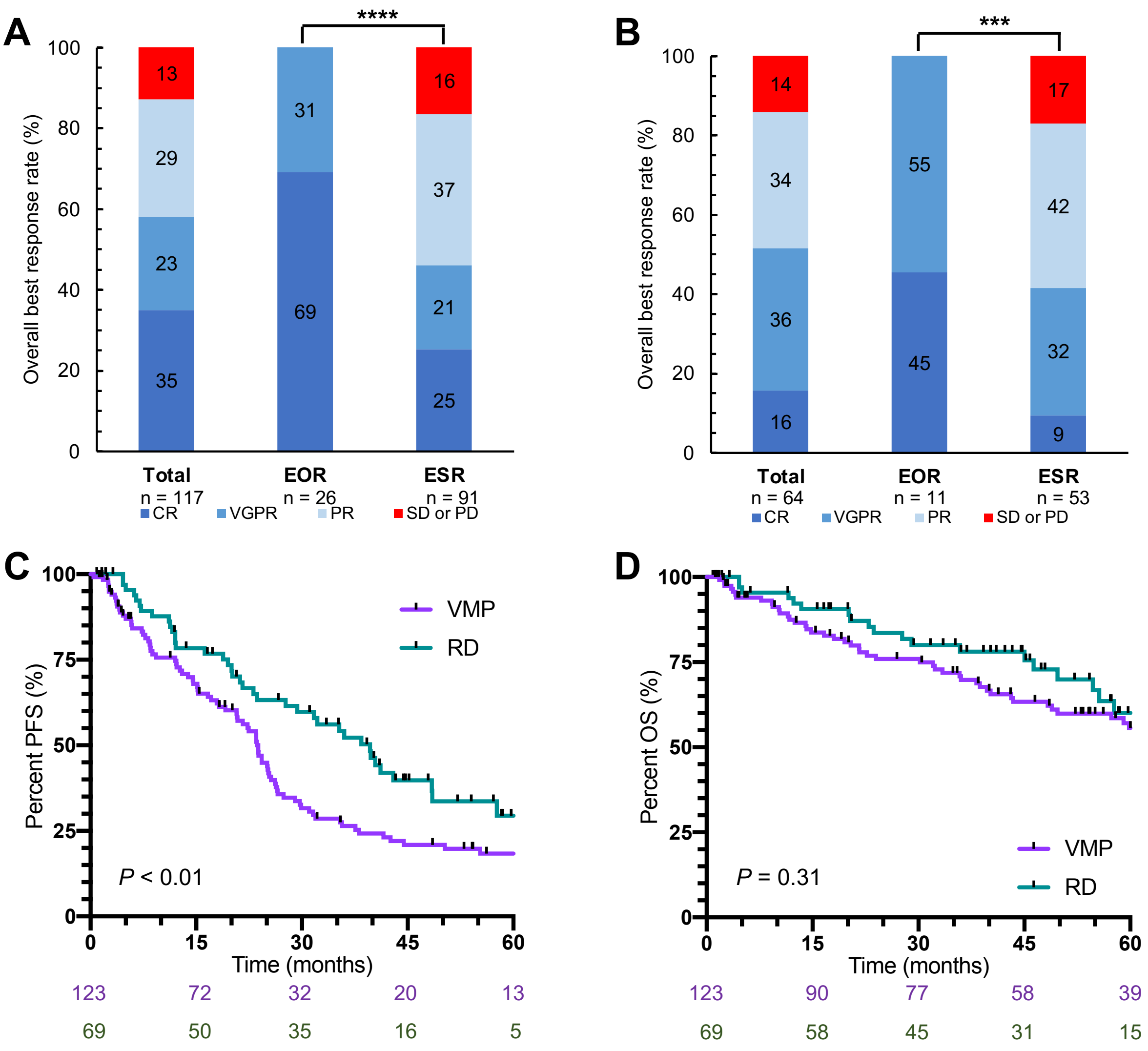

Supplementary Fig. 7. Clinical outcome and survival of the external test cohort (MMRF & SNU, N = 192). Overall response rates of the NDMM patients to (**A**) VMP or (**B**) RD regimen as the first-line treatment. The *P* values are calculated using the Fisher's exact test. (**C**) PFS and (**D**) OS of the VMP or RD treated NDMM patients. The *P* values are calculated by the log-rank test.

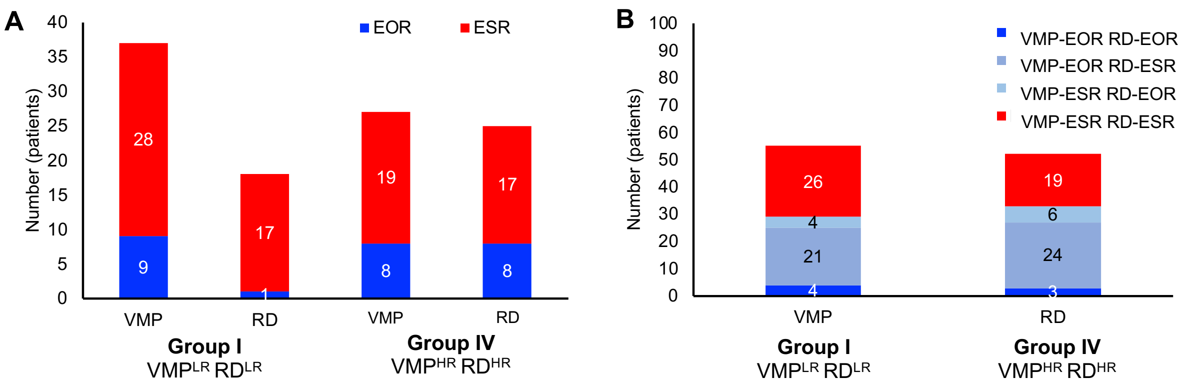

Supplementary Fig. 8. ML response models and its application to further refine treatment for the Group I and IV of the test cohort. (A) Proportions of the EOR and ESR in the Group I and IV based on the conventional treatment selection. (B) Predicted proportions of the EOR and ESR to VMP and RD based on the ML response models

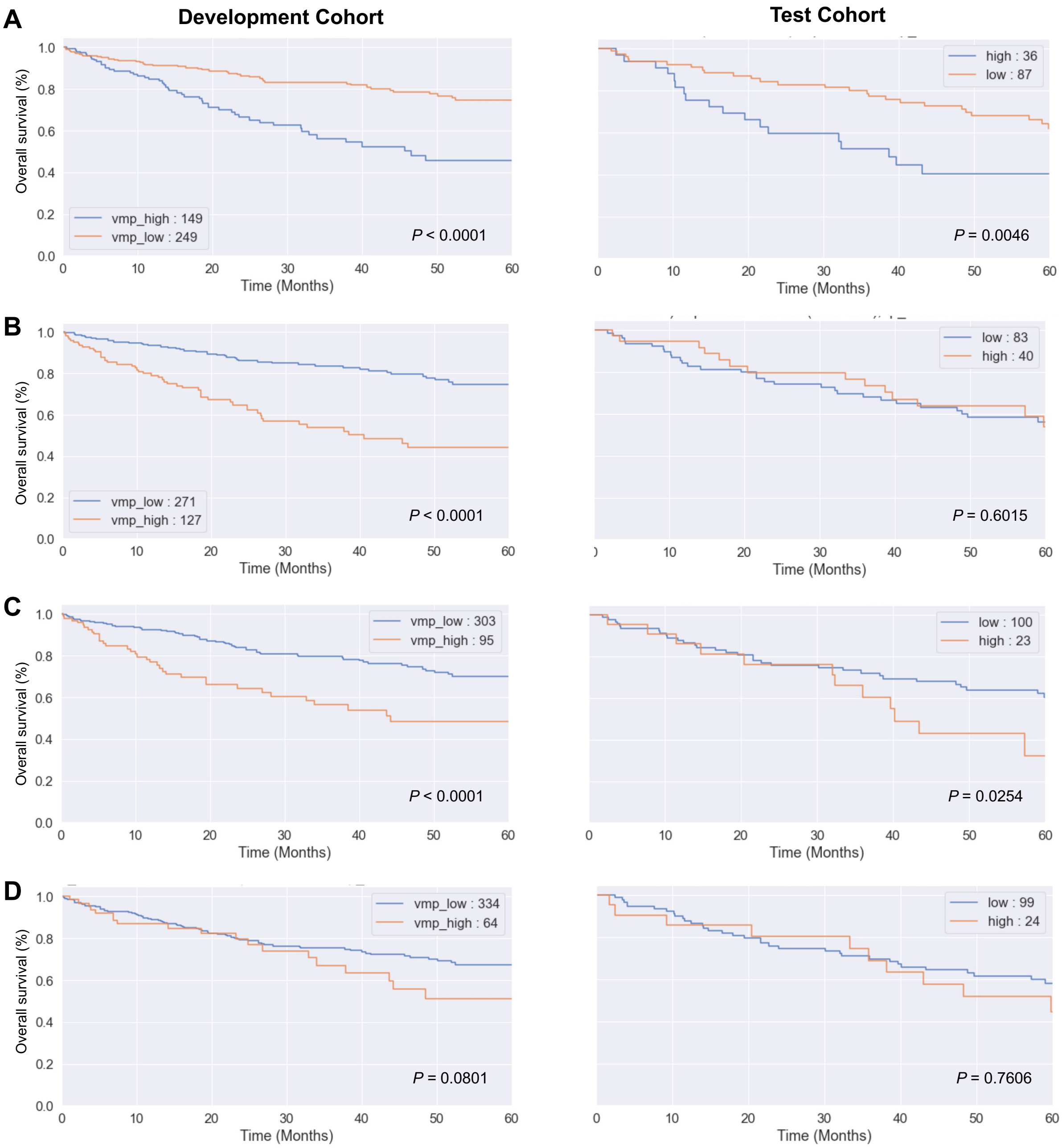

Supplementary Fig. 9. Changes in the predictive performance of the ML survival models with respect to the handling of missing values. OS of the resultant risk stratification when the missing values were (**A**) left as is, (**B**) replaced with median, imputed using (**C**) kNN, or (**D**) MICE.

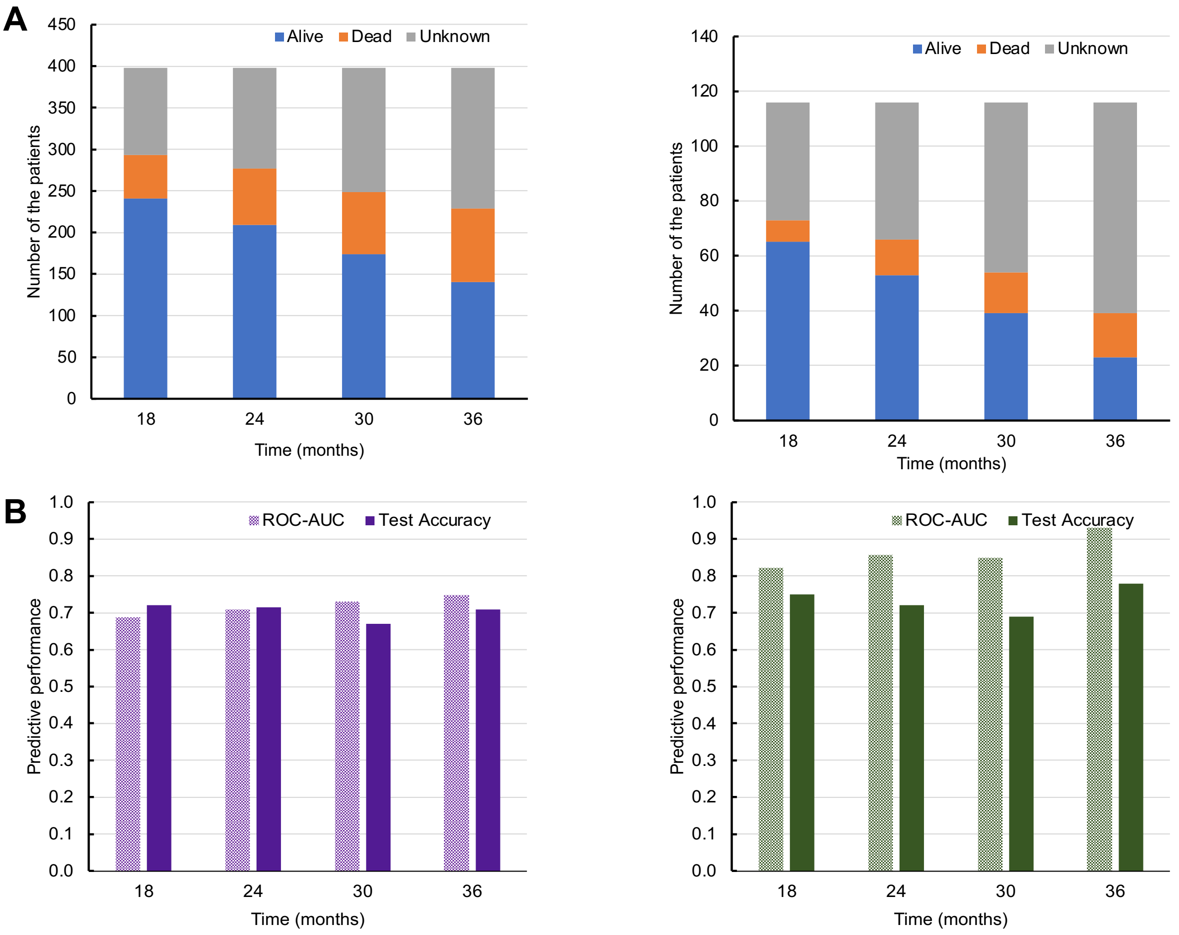

Supplementary Fig. 10. Changes in the (A) ratio of dead to alive and (B) predictive performance of the ML survival models (VMP left, RD right) with respect to the time point chosen to stratify the patients into risk groups. The number of patients shown in (A) are about the development cohort used to train the models. In terms of the predictive performance, ROC-AUC were measured during validation while test accuracy was assessed using the test cohort.

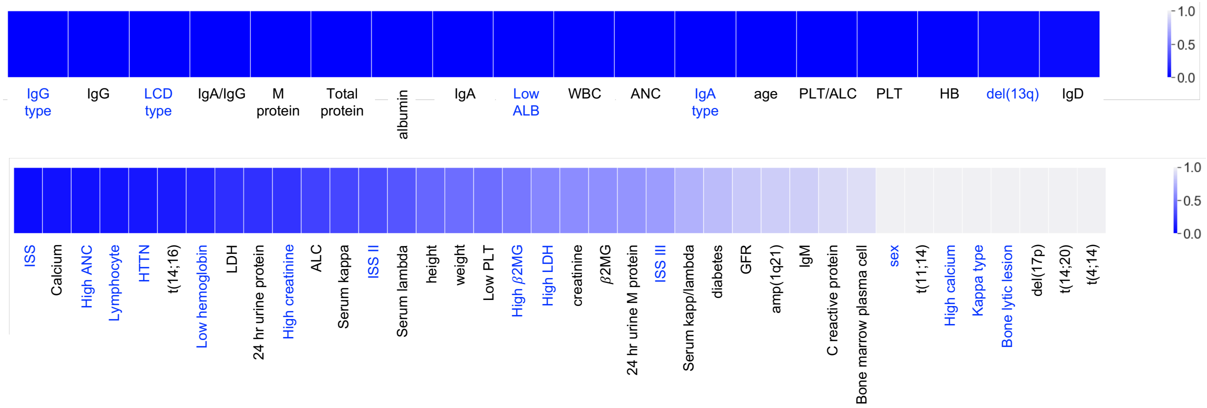

Supplementary Fig. 11. Ranked heat map of the *P* values obtained when comparing the EOR and ESR to VMP regimen. The clinical characteristics in the upper rows are the ones that recorded $P < 0.05$ between the two subgroups. LCD, light chain disease; WBC, white blood cell; ANC, absolute neutrophil count; PLT, platelet count; HB, hemoglobin level; HTTN, hypertension; LDH, lactate dehydrogenase level; UEP, urine proteins level; ALC, absolute lymphocyte count; Wt, weight; β2MG, β2 microglobulin level; CREA, creatine level; DM, diabetes; GFR, glomerular filtration rate. High ANC, ANC > 1.0 x 10^9/L; low hemoglobin, hemoglobin < 10.0 g/dL; high creatinine, creatinine > 2.0 mg/dL; low PLT, PLT < 75 x 10^9/L; high β2MG > 5.5 μg/mL; high LDH, LDH > upper normal limit; high calcium, calcium > 11.0 mg/dL. Categorical covariates are written in blue while the numerical ones are written in black.
