# Supplementary material for: ML-based sequential analysis to assist selection between VMP and RD for newly diagnosed multiple myeloma
Source: NPJ Precis Oncol. 2023 May 20;7:46. doi: 10.1038/s41698-023-00385-w (PMC10199943; doi:10.1038/s41698-023-00385-w)
Supplement: Supplementary file 2 — REPORTING SUMMARY [file 41698_2023_385_MOESM2_ESM.pdf]

nature portfolio

Corresponding author(s): Jamin Koo, Chang-Ki Min

Last updated by author(s): Feb 15, 2023

Reporting Summary

Nature Portfolio wishes to improve the reproducibility of the work that we publish. This form provides structure for consistency and transparency in reporting. For further information on Nature Portfolio policies, see our Editorial Policies and the Editorial Policy Checklist.

Statistics

For all statistical analyses, confirm that the following items are present in the figure legend, table legend, main text, or Methods section.

n/a	Confirmed	
☐	☒	The exact sample size (*n*) for each experimental group/condition, given as a discrete number and unit of measurement
☐	☒	A statement on whether measurements were taken from distinct samples or whether the same sample was measured repeatedly
☐	☒	The statistical test(s) used AND whether they are one- or two-sided<br>*Only common tests should be described solely by name; describe more complex techniques in the Methods section.*
☐	☒	A description of all covariates tested
☐	☒	A description of any assumptions or corrections, such as tests of normality and adjustment for multiple comparisons
☐	☒	A full description of the statistical parameters including central tendency (e.g. means) or other basic estimates (e.g. regression coefficient) AND variation (e.g. standard deviation) or associated estimates of uncertainty (e.g. confidence intervals)
☐	☒	For null hypothesis testing, the test statistic (e.g. *F*, *t*, *r*) with confidence intervals, effect sizes, degrees of freedom and *P* value noted<br>*Give P values as exact values whenever suitable.*
☒	☐	For Bayesian analysis, information on the choice of priors and Markov chain Monte Carlo settings
☒	☐	For hierarchical and complex designs, identification of the appropriate level for tests and full reporting of outcomes
☐	☒	Estimates of effect sizes (e.g. Cohen's *d*, Pearson's *r*), indicating how they were calculated

Our web collection on statistics for biologists contains articles on many of the points above.

Software and code

Policy information about availability of computer code

Data collection	The clinical data used in this study were extracted from the multi-center registry database of the Catholic Research Network for Multiple Myeloma (CARE-MM), which include four university hospitals in Republic of Korea. The independent test datasets, to test the ML models trained in the development cohort, camewere obtained from the two different sources: CoMMpass database (version IA17) and Seoul National University (SNU) Hospital. The datasets were provided in Excel or csv file formats.
Data analysis	We used python and/or Prism to analyze data and build machine learning models. The specific packages used to conduct analysis are as follows: InStat (Prism), roc_curve, auc package (scikit-learn), and sksurv (Python).

For manuscripts utilizing custom algorithms or software that are central to the research but not yet described in published literature, software must be made available to editors and reviewers. We strongly encourage code deposition in a community repository (e.g. GitHub). See the Nature Portfolio guidelines for submitting code & software for further information.

Data

Policy information about availability of data

All manuscripts must include a data availability statement. This statement should provide the following information, where applicable:

- Accession codes, unique identifiers, or web links for publicly available datasets
- A description of any restrictions on data availability
- For clinical datasets or third party data, please ensure that the statement adheres to our policy

The datasets generated and/or analyzed during the current study are available from the corresponding author on reasonable request.

Human research participants

Policy information about studies involving human research participants and Sex and Gender in Research.

Reporting on sex and gender	The median age was 70 and ranged from 40 to 92. Half of the patients were male while the other half were female. Further description about patient demographics can be found in Table 1.
Population characteristics	All patients of the CARE-MM cohort used to train the ML models were Asian. The majority of the MMRF cohort were white (83%) or black (15%) while the SNU cohort were all Asian. The median age at the time of diagnosis was similar among the three cohorts. The median OS of the patients treated with VMP or RD regimen in the CARE-MM cohort were 95 and 100 months, respectively; the values were 67 and 62 months among the test cohort (MMRF and SNU) for the two regimens. The overall response rates were 86 vs 91% for the VMP and RD regimen; the rates were similar among the test cohort (87 vs 86%). The median PFS of the patients treated with VMP or RD regimen were 15 and 21 months, respectively. The PFS lasted 24 or 40 months for the NDMM in the test cohort treated by the two regimens. More information are provided in Table 1.
Recruitment	The clinical data used in this study were extracted from the multicenter registry database of the Catholic Research Network for Multiple Myeloma (CARE-MM), which include four university hospitals in Republic of Korea. There were records of the 1359 consecutive NDMM patients who received the novel agent based-therapy as the first-line treatment between June 2010 and July 2021. Among them, 786 patients treated with the options other than VMP or RD regimen were excluded. We further excluded 59 patients who did not have the baseline data or response outcome (Fig. 1A).
Ethics oversight	Catholic University, Seoul National University

Note that full information on the approval of the study protocol must also be provided in the manuscript.

Field-specific reporting

Please select the one below that is the best fit for your research. If you are not sure, read the appropriate sections before making your selection.

☒ Life sciences ☐ Behavioural & social sciences ☐ Ecological, evolutionary & environmental sciences

For a reference copy of the document with all sections, see nature.com/documents/nr-reporting-summary-flat.pdf

Life sciences study design

All studies must disclose on these points even when the disclosure is negative.

Sample size	The development and test cohorts were 514 and 192 in number. These are the largest sizes we have seen in literature when applied the same inclusion and exclusion criteria.
Data exclusions	The clinical data used in this study were extracted from the multicenter registry database of the Catholic Research Network for Multiple Myeloma (CARE-MM), which include four university hospitals in Republic of Korea. There were records of the 1359 consecutive NDMM patients who received the novel agent based-therapy as the first-line treatment between June 2010 and July 2021. Among them, 786 patients treated with the options other than VMP or RD regimen were excluded. We further excluded 59 patients who did not have the baseline data or response outcome (Fig. 1A).
Replication	We used the independent dataset of 192 NDMM patients to further validate the proposed machine learning models.
Randomization	This is not relevant to the proposed study as we are not comparing treatments but training models to predict response to treatments.
Blinding	Blinding is also not relevant due to the same reasons as stated in the above.

Reporting for specific materials, systems and methods

We require information from authors about some types of materials, experimental systems and methods used in many studies. Here, indicate whether each material, system or method listed is relevant to your study. If you are not sure if a list item applies to your research, read the appropriate section before selecting a response.

Materials & experimental systems

n/a	Involved in the study
☒	☐ Antibodies
☒	☐ Eukaryotic cell lines
☒	☐ Palaeontology and archaeology
☒	☐ Animals and other organisms
☐	☒ Clinical data
☒	☐ Dual use research of concern

Methods

n/a	Involved in the study
☒	☐ ChIP-seq
☒	☐ Flow cytometry
☒	☐ MRI-based neuroimaging

Clinical data

Policy information about clinical studies

All manuscripts should comply with the ICMJE guidelines for publication of clinical research and a completed CONSORT checklist must be included with all submissions.

Clinical trial registration	Not applicable. It is a retrospective study.
Study protocol	Not applicable.
Data collection	The clinical data used in this study were extracted from the multicenter registry database of the Catholic Research Network for Multiple Myeloma (CARE-MM), which include four university hospitals in Republic of Korea. There were records of the 1359 consecutive NDMM patients who received the novel agent based-therapy as the first-line treatment between June 2010 and July 2021. Among them, 786 patients treated with the options other than VMP or RD regimen were excluded. We further excluded 59 patients who did not have the baseline data or response outcome (Fig. 1A).
Outcomes	OS and early optimal response were the clinical endpoints for which predictive ML models were developed. None were used as features in training the ML models. The index date of both endpoints was the first day of administration of VMP or RD. The former was measured from the index date to censoring or death from any cause. The latter was defined as the achievement of complete response (CR) or very good partial response (VGPR) by the 8th week since the index date; partial response, stable disease, or refractory disease were classified as early suboptimal response. Response to each VMP or RD was evaluated using the International Myeloma Working Group's (IMWG's) response criteria [24]. Progression-free survival (PFS) was calculated as the durations from the index date until progression or death.
